# Sounds, Behaviour, and Auditory Receptors of the Armoured Ground Cricket, *Acanthoplus longipes*

**DOI:** 10.1673/031.010.5901

**Published:** 2010-06-10

**Authors:** Kerstin Kowalski, Reinhard Lakes-Harlan

**Affiliations:** Institute for Animal Physiology, Justus-Liebig University Giessen, Wartweg 95, 35392 Giessen, Germany

**Keywords:** Calling song, courtship behaviour, disturbance sound, hearing system, scolopidial organ, sensory physiology

## Abstract

The auditory sensory system of the taxon Hetrodinae has not been studied previously. Males of the African armoured ground cricket, *Acanthoplus longipes* (Orthoptera: Tettigoniidae: Hetrodinae) produce a calling song that lasts for minutes and consists of verses with two pulses. About three impulses are in the first pulse and about five impulses are in the second pulse. In contrast, the disturbance stridulation consists of verses with about 14 impulses that are not separated in pulses. Furthermore, the inter-impulse intervals of both types of sounds are different, whereas verses have similar durations. This indicates that the neuronal networks for sound generation are not identical. The frequency spectrum peaks at about 15 kHz in both types of sounds, whereas the hearing threshold has the greatest sensitivity between 4 and 10 kHz. The auditory afferents project into the prothoracic ganglion. The foreleg contains about 27 sensory neurons in the crista acustica; the midleg has 18 sensory neurons, and the hindleg has 14. The auditory system is similar to those of other Tettigoniidae.

## Introduction

The acoustic system plays an important role in communication and behaviour of orthopteran insects. This auditory communication system can be divided into the sound production organs and the sound perceiving organs, as well as their neuronal processing systems. Acoustic signals are used for intraspecific communications but also for interspecific interactions (Alexander 1967; Ewing 1989; Bailey 1990; Gerhardt and Huber 2002; Greenfield 2002). Most species of Tettigoniidae use acoustic communication, and consequently, it is relatively well studied (Bailey and Rentz 1990). However, the taxon Hetrodinae has received little attention in this respect, despite its potential importance for biological control of pest species.

One main function of the intraspecific auditory communication between females and males is to assist pair formation (Robinson 1990). Therefore these acoustic signals are stereotypical with a distinct structure for a given species. The temporal pattern and frequency components of these songs are species specific and are widely used for taxonomy and ecological analysis (Heller 1988; Ragge and Reynolds 1998; Walker et al. 2003; Elliott and Hershberger 2007).

Another type of acoustic signal that is used in many insect taxa (e.g. Coleoptera (Lewis and Cane 1990; Schilman et al. 2001) and Homoptera (Stölting et al. 2004)), is the disturbance sound. These alarm signals are made by insects disturbed in different manners e.g. by touching. In contrast to the calling song, the disturbance sound has a simple and irregular temporal pattern (Masters 1980). Alexander (1967) reported that arthropods use the sound production for a defensive mechanism more often than for any other acoustical communication.

The ear of Tettigoniidae is located in the proximal area of the foreleg tibia (Graber 1876; Schumacher 1979). The scolopidial cells, specialized for detecting mechanical forces, show a typical arrangement in the proximal tibia of Tettigoniidae (Schumacher 1973; Lakes and Schikorski 1990). These cells form a complex tibial organ, consisting of the subgenual organ, the intermediate organ, and the crista acustica; the latter perceives airborne sound (Stumpner 1996). The auditory fibres run from the tibial organ through nerve 5B1 into the prothoracic ganglion where they terminate in the auditory neuropile (Römer et al. 1988).

The Hetrodinae are distributed all over Africa and neighbouring areas (Grzeschik 1969; Irish 1992) and are called armoured ground (or bush) crickets because of spikes on their pronotum and legs. These bush crickets are flightless with rudimentary wings that are covered under the pronotum (Weidner 1955). *Acanthoplus longipes* (Orthoptera: Tettigoniidae: Hetrodinae) is a dark brown and ventrally green bushcricket with spines only on the pronotum. They are sexually dimorphic, and males use an elytroelytral stridulatory mechanism, as is the case with most bushcrickets. *A. longipes* lives in the low grassland of Southwest Africa (Namibia, Angola, and Congo) where it can have plague status in field crops when its population climaxes between March and May (Weidner 1955; Mbata 1992). The importance for agricultural ecosystems leads to investigations about the reproductive system of *Acanthoplus* spp. (Mbata 1992; Bateman and Ferguson 2004). The acoustic system of Tettigoniidae is an important part of the reproductive system. In respect to the auditory system it has been shown that *Acanthoplus* spp. have a pulsed calling song (Conti and Viglianisi 2005), but the sensory organs have not been investigated. Therefore, the acoustic signals, as well as the anatomy and physiology of the sound receiver, are described.

## Materials and Methods

### Bushcrickets

**Figure 1.**
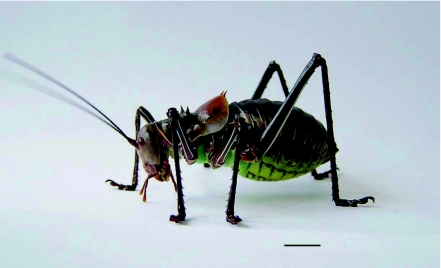
Photograph of a male *Acanthoplus longipes*. Scale: I cm, relative to the pronotum. High quality figures are available online.

*A. longipes* (Figure 1) were collected as nymphs on roads near Keetmanshoop (26° 32′ S, 18° 6′ E), Namibia in March 2008 and transferred to the University of Giessen. The species was identified based on the key from Irish (1992). Four female and seven male *A. longipes* were used for the experiments. The animals were sorted by sex and kept between 22° C and 30° C with a 12: 12 lightdark cycle. They were fed with wheat seedlings, dog and fish food, and water *ad libitum*.

### Sound recordings and analysis

For the sound recordings, the bushcrickets were placed within a cage of fly-screen in an anechoic chamber (50 × 50 × 50 cm). Each of six males was recorded once.

The recordings of the calling song were made in the dark, while the recordings of the disturbance stridulation were made under light conditions. To evoke a disturbance sound, the resting insects (*n* = 2) were briefly touched with a stick. The songs were recorded at a temperature between 23° C and 27° C. An ultrasound microphone (Ultra Sound Gate CPVS, Avisoft Bioacoustics, www.avisoft.com) with a frequency range of 10 to 95 kHz connected to a digital recorder (Tascam HD-P2) with a sampling rate of 192 kHz was used. The microphone was placed 15 to 40 cm away from the bushcrickets. Sound pressure level was measured with a Voltcraft meter (DT-8820). Both temporal structure and frequency range of the recordings were analyzed on a computer with the AviSoft program. For statistical analysis, Prism 4.03 (GraphPad Software, Inc., www.graphpad.com) was used. The following terminology was used for describing the insect sounds:

Impulse: A single impulse probably caused by movement of one tooth of the stridulatory file.

Pulse: A train of impulses which are produced by opening or closing the wings. Verse: A group of impulses, which can contain one or two pulses.

For the analysis of the courtship behaviour, four virgin female *A. longipes* were tested. For each test, one female and one male were put together into a terrarium.

### Hearing threshold

For electrophysiological investigations, *A. longipes* (*n* = 5) were waxed on a metal holder with the ventral side up, and the forelegs were fixed approximately in their natural position. The hindlegs were removed and the midlegs were fixed with wax. The prothorax was opened ventrally and the prothoracic ganglion, the leg nerve, and the tympanal nerve were exposed. The recordings were made extracellularly from the tympanal nerve close to the bifurcation from the leg nerve. The tympanal nerve was put on a silver wire electrode, and the indifferent electrode was inserted contralaterally in the thorax. The signals from the nerve were amplified 1.000x by a preamplifier (T122, Tektronix, Inc., www.tek.com), visualized on an oscilloscope, and connected to earphones. The sound signals were computer generated and amplified. They were made audible by a loudspeaker (SEAS 11 F-GX), which was positioned laterally 38 cm from the insect. The tested frequencies ranged from 3 to 40 kHz and were played back with sound pressure levels from 30 to 80 dB. Each sound intensity was tested five times. The lowest acoustic stimulus which elicited neuronal responses was defined as the auditory threshold.

### Neuroanatomy

For the anatomical studies of the periphery, all legs (of 7 *A. longipes*) were removed and placed into Petri dishes filled with saline solution. The legs were opened proximally at the femur-tibia joint, and the tympanal nerve (N5B1) was cut and placed in a glass capillary filled with 5% cobalt chloride solution in distilled water. Preparations were placed in a moist chamber for two days at 4° C. The staining was visualized with a 1% solution of ammonium sulphide in phosphate buffer. The legs were fixed in 4% of paraformaldehyde, dehydrated in a graded ethanol series, and cleared in methylsalicylate. As it was not possible to see the scolopidial cells through the dark cuticle, the tibia was opened dorsally by careful dissection.

For the anatomical studies of the central nervous system, the prothoracic ganglion was removed from the animal and placed in a Petri dish. The tympanal nerve (N5B1) was placed in a glass capillary, which was filled with a 5% neurobiotin solution in distilled water. The preparation was incubated at 4° C in a moist chamber for 48 hours. Thereafter, the ganglion was fixed in 4% paraformaldehyde. Then it was dehydrated, cleared in xylene for 5 minutes, and rehydrated. The next step was incubation in collagenase and hyaluronidase solution (1 mg each, Sigma Chemicals, www.sigmaaldrich.com) in 1 ml phosphate buffer for one hour at 37° C. The ganglion was placed in an Avidin-Biotin-Complex (Vectastain ABC Kit PK-6100 Vector Laboratories, www.vectorlabs.com) over night. After washing with phosphate buffer, the marking was visualized with DAB and H_2_O_2_ (Vector Peroxidase Substrate Kit DAB SK-4100, Vector Laboratories) under visual control. The ganglion was dehydrated and cleared in methylsalicylate.

All preparations were documented by drawings (Leitz Dialux microscope with a drawing tube) and photographs (Olympus BH-2 microscope, www.olympus.com, with a Leica DCF-320 camera, www.leicamicrosystems.com).

## Results

### Sound of *A. longipes*

**Figure 2.**
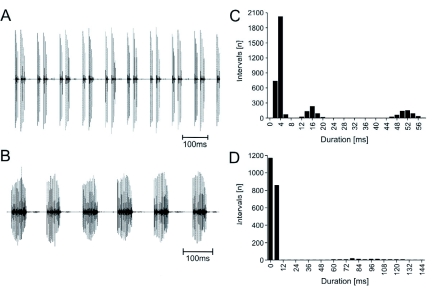
Oscillogram of the calling song (A) and disturbance stridulation (B) with a quantification of the interval duration of the sound types. (C) Occurrence of different interval durations in the calling song (Bin width 2 ms; n = 3702). (D) Occurrence of different interval durations in the disturbance sound (Bin width 6 ms; *n* = 2155). High quality figures are available online.

The calling song of *A. longipes* males (Figure 2A) was produced in the late evening. The males were persistent singers, often singing for several minutes without any interruptions. Most stayed in one place, usually elevated, while singing, but some walked around without stopping to sing. The sound pressure level reached about 87 dB SPL in a distance of 10 cm caudal (*n* = 4).

**Figure 3.**
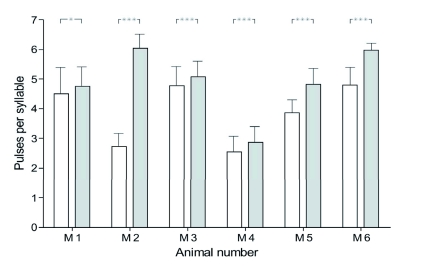
Impulse number per pulse in the calling song of six different male *Acanthoplus longipes* (M1–M6). Open bars represent the impulses in the first pulse, grey bars represent the impulses of the second pulse; means with standard deviation; *n* (per verse) of each male = 100; unpaired t-test (first and second pulse); M1 : p < 0.05, M2–M6: ñ < 0.001; the songs were recorded at 23–27° C. High quality figures are available online.

**Figure 4.**
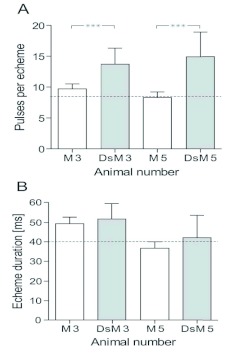
Verse duration (A) and impulses per verse (B) in the calling song and disturbance sound of two male *Acanthoplus longipes* (M3 and M5). Open bars (M3 and M5) represent the calling songs and grey bars represent the disturbance stridulation (DsM3 and DsM5). The dotted lines show the means of the calling song in all investigated males (*n* = 6); values are presented as means with standard deviation; unpaired t-test (calling song and disturbance sound); M3 and M5: p < 0.0001; *n* (M3) = 471, *n* (M5) = 794, *n* (DsM3) = 80, *n* (DsM5) = 154. High quality figures are available online.

The calling song consisted of a sequence of verses that were separated into two pulses by a pause of about 16 ms (Figures 2A, 2C). These two pulses consisted of 2 to 7 impulses, which differed between the tested males (Figure 3), but all males had fewer impulses in the first pulse than in the second pulse. In one of the six males (M2 in Figure 3) the second pulse more than doubled the number of impulses in the first pulse. The impulse interval (3.5 ms, *n* = 2811; SD = 0.68) was similar in the first and the second pulses (Figure 2C), which were separated by an interpuise interval of about 16 ms. The verse interval was about 50 ms. The mean verse duration was 40 ms (Figure 4A), and the mean number of impulses per verse was 8.53 pulses (Figure 4B).

Disturbance stridulation (Figure 2B) could be more easily elicited during the day than during the night and from resting insects than from walking insects. For two males, recordings from both types of sounds were compared (Figure 4). The disturbance sound showed three characteristic differences to the calling song. First, the disturbance stridulation lasted only a few seconds. Second, the disturbance stridulation consisted of verses with only one pulse. Third, the pulses consisted of about 13 or 14 impulses per verse in contrast to the maximum number of 10 impulses per verse in the calling song (Figure 4A). The mean number of impulses per verse between the calling song and the disturbance stridulation was significantly different (Figure 4; unpaired t-test; p < 0.0001; t = 45.45; df = 1495; calling song: *n* = 1262; disturbance sound: *n* = 235) in both males. However, the duration of the verses of both sounds was not different (Figure 4B). The sound pattern resulted in two groups of interval durations (Figure 2D). The verse interval was rather variable (mean = 98 ms; *n* = 220; SD = 62.50), but the impulse interval (2.9 ms; *n* = 2028; SD = 0.78) was invariant and significantly different from that of the calling song (p < 0.0001, unpaired T-test, dft = 26.13, df= 4837).

**Figure 5.**
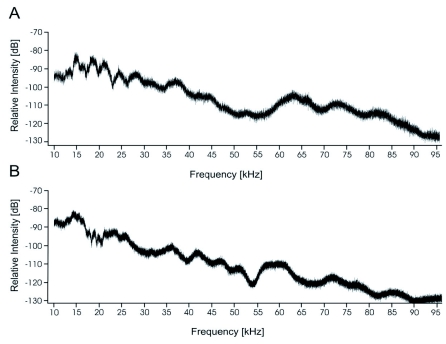
Frequency spectrum above 10 kHz of the disturbance sound (A) and the calling song (B) of the same male *Acanthoplus longipes* (M5). High quality figures are available online.

**Figure 6.**
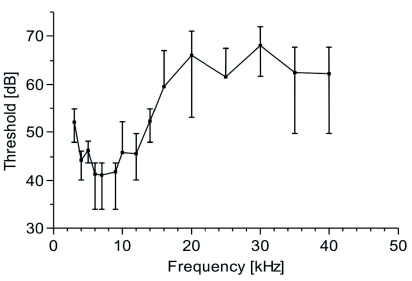
Hearing threshold of *Acanthoplus longipes* from extracellular recordings of the tympanal nerve; means with standard deviation; *n* = 5 (male = 2; female = 3). High quality figures are available online.

Both types of songs had similar frequency spectra within the investigated range with a peak around 15 kHz and a steady decrease in the ultrasonic range (Figure 5).

### Defense behaviour

Disturbance stridulation can be regarded as one mechanism of defense. While producing the sound, *A. longipes* always started to run away. As an additional defense mechanism, both sexes used reflex bleeding. They extruded hemolymph liquid from the coxa-trochanter joint. The squirt intensity and the bleeding coxa-trochanter joints could vary. The bleeding could not be elicited by a brief touch, but by handling the insects, e.g. during preparation for experiments. Otherwise, no complex defense mechanisms were observed.

### Courtship

Females performed positive phonotaxis toward singing males. Whereas 3 of 4 females paused during phonotaxis, 1 female approached the male very quickly. When females reached the males, they touched them with their long antennae, and the males stopped singing. All observed pairs met each other under the top of the cage, and the male climbed underneath the female from a lateral position. Mating only started in the late evening and took at least 2 hours. On the next morning, 3 of 4 females still carried the spermatophore. One spermatophore was removed and weighted: 0.46 g, 5.4% of the respective male's weight. Females were heavier (mean 11.6 g, *n* = 3) than the males (mean 8.5 g, *n* = 2). During the day, the females fed on the spermatophore. For egg laying, the female, with its abdomen, made a small hole in the sand and placed a cluster of eggs into it.

**Table 1:**
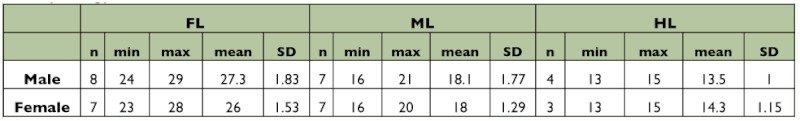
Number of receptor cells in the crista acustica in the foreleg (FL), midleg (ML) and hindleg (HL) of male and female *Acanthoplus longipes*.

### Electrophysiology

The hearing threshold showed the highest sensitivity from 4 and 10 kHz with a threshold between 40 and 45 dB SPL (Figure 6). The threshold rose to about 60 dB SPL in the ultrasonic range (20 – 40 kHz). No differences between males and females were found.

### Neuroanatomy

**Figure 7.**
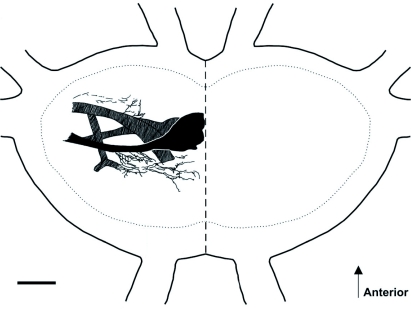
Drawing of the central projection of the nerve 5B1 in the prothoracic ganglion of *Acanthoplus longipes*. Black: projection of auditory fibres; grey: projections of other sensory cells within the tympanal nerve; scale: 500 µm. High quality figures are available online.

The anterograde backfills of the tympanal nerve into the prothoracic ganglion showed that the nerve, 5B1, projects through the leg nerve. The axonal fibres of the auditory receptors continued in a posterior curve to the midline of the ganglion and terminated ipsilaterally in a dense neuropile (Figure 7).

**Figure 8.**
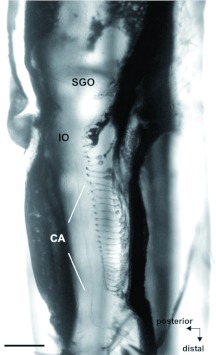
Dorsal view of the complex tibial organ in the foreleg of an *Acanthoplus longipes* female. The sensory neurons of the crista acustica (CA), the intermediate organ (10) and the subgenual organ (SGO) are labelled by a nerve backfill with cobalt chloride. Scale: 500 µm. High quality figures are available online.

Peripheral backfills into the tibia showed the typical tripartite organization of the sensory complex for Tettigoniidae: subgenualorgan, intermediate organ, and crista acustica (Figure 8). *A. longipes* had about 27 neurons in the crista acustica of the foreleg, 18 cells in the midleg, and 14 crista acustica neurons in the hindleg (Table 1), with no sexual dimorphism.

## Discussion

### Calling song and courtship of *A. longipes*

The calling song of *A. longipes* is a sequence of two pulse verses, which can last several minutes. Each verse consists of two pulses, which consist of a few impulses. The impulse numbers in the pulses vary among individuals (see also Conti and Viglianisi 2005). The songs often show some variations within a basic pattern (Schul 1998), which could be important for sexual selection. Larger variation might raise a problem when females need an exact pattern of the calling song to recognize the species-specific song (Klappert and Reinhold 2003), which is the case with females from areas of sympatry (Gwynne 2001). Variable song pattern could lead to heterospecific mating in closely related species, as has been shown for Acrididae (von Helversen and von Helversen 1975).

The results on the frequency spectrum extend those of Conti and Viglianisi (2005) in the ultrasonic range and confirm a broad peak between 10 and 15 kHz. This frequency spectrum lies within the range of other Tettigoniidae (Heller 1988; Römer et al. 1989; Schul and Patterson 2003). The fact of frequency attenuation of the vegetation, especially for the ultrasonic components of the calling song (Keuper et al. 1986; Römer and Lewald 1991), might be the reason that *A. longipes* males seemed to prefer singing from a higher position. This has to be confirmed by field studies.

The auditory threshold shows the greatest sensitivity to between 4 and 10 kHz, which reflects a mismatch to the frequency spectrum of the calling song. In other Tettigoniidae, a species-specific tuning to the song spectrum is found, although the temporal pattern might be even more important (Dobler et al. 1994; Römer and Bailey 1998; Schul and Patterson 2003; Lehmann et al. 2007). Phonotaxis experiments with song models could clarify how species recognition in *A. longipes* is influenced by song frequency or by song pattern. In the laboratory, no chorusing of *A. longipes* could be observed, as was observed in the Hetrodinae *Acanthoplus speiseri* (Mbata 1992) and *Eugaster* spp. (Grzeschik 1969). This shows a considerable variation of acoustic signalling in a genus similar to other Tettigoniidae (Greenfield et al. 2004; Fertschai et al. 2007).

The phonotactic behaviour of the females, which is a common reaction to the conspecific calling song in tettigoniids, is also described for other *Acanthoplus* species (Power 1958) and for *Eugaster* species (Weidner 1955; Grzeschik 1969). However, no courtship song could be observed for *A. longipes* as in *A. speiseri* (Mbata 1992) and *Eugaster* spp. (Grzeschik 1969). The mating and egg laying behaviour is similar to those of other Hetrodinae species (Weidner 1955; Power 1958; Grzeschik 1969; Mbata 1992) although the mating duration seems to be much longer.

### Disturbance sound and defense

The disturbance sounds of orthopterans are less well studied (Field 1993; Desutter-Grandcolas 1998). Some Tettigoniidae use their stridulation mechanism for both intraspecific communication and as a defense mechanism (Kaltenbach 1990), and other species use different organs for disturbance stridulation (Heller 1996). Additionally, other defensive behaviour with and without sound production evolved (Belwood 1990). The disturbance stridulation of *A. longipes* could be evoked by disturbing resting animals. It was a brief sound that stopped shortly after the disturbance. The verses consist of more impulses that are not separated into pulses, compared to the calling song. The verse interval is variable; thus, the rather plain and variable pattern fits two of four characteristics of a disturbance sound (simple and irregular) proposed by Masters (1980). The two other characteristics (broad frequency band and a maximum energy at 1 kHz) could not be found in the disturbance stridulation of *A. longipes*. The frequency spectrum of the disturbance stridulation and the calling song are similar. It has been found for other orthopterans, as well, that the four characteristics do not always fit to disturbance sounds (Desutter-Grandcolas 1998). The different impulse interval together with the different verse structure indicate that the disturbance stridulation does not simply reflect the neuronal and functional networks involved in calling song stridulation.

Many species who use disturbance sounds are large, flightless, slow-moving and night-singing bushcrickets, for example *Pterophylla camellifolia, Liparoscelis nigrispina* and *Aglaothorax armiger* (Alexander 1960), which leads some authors to the assumption that this kind of sound production is a defense mechanism, especially against vertebrates (Alexander 1967; Belwood 1990). The disturbance stridulation might increase the chance of survival of an insect after a predatory attack because it might startle the predator (Robinson and Hall 2002). Or it might have a warning function for an additional defense mechanism, e.g. noxious signals (Masters 1979). Furthermore, it is possible that a defense sound is mimicking an aposematic signal. While camouflage and mimicry are primary defense mechanisms (Gwynne 2001), the disturbance stridulation is a secondary defense mechanism, which is used after the predator has made contact with the potential prey. There are also some arguments against the hypothesis of a defense mechanism: if this type of sound is an important defense mechanism both sexes should be able to produce it (Heller 1996). Only in tettigoniid species, where the females also produce a sound for intraspecific communication, both sexes produce disturbance sounds (Shaw and Galliart 1987). Furthermore, nymphs should also benefit from such a defense mechanism, as in some tettigoniid species (Dadour and Bailey 1990).

*A. longipes* showed no complex behavioural pattern for defense, as other orthopterans do (Sandow and Bailey 1978), but like other Hetrodinae (Weidner 1955; Power 1958; Grzeschik 1969), both sexes use reflex bleeding as an additional, secondary defense mechanism. However, there is no evidence that the hemolymph of *A. longipes* is noxious. Additionally, *A. longipes* is well armed with spines, making it a difficult prey for small animals. The complement of different defense mechanisms might be necessary for day-active, ground-living flightless animals that otherwise might become an easy prey.

### Neuroanatomy of the auditory system

Retrograde backfills of the legs show a complex of scolopidial cells in the proximal tibia, which can be divided into three parts. The most proximal group of cells is the subgenual organ, which detects substrate vibrations. The middle part is the intermediate organ, and the third part is the crista acustica, which perceives airborne sound (Stumpner 1996). This complex tibial organ can be found in all legs, although tympana are only present in the foreleg. In the crista acustica, the cell number is species-specific and ranges between 20 and 50 cells in different species of the Tettigoniidae (Schumacher 1979; Lakes and Schikorski 1990; Kalmring et al. 1993; Robinson and Hall 2002). The number of crista acustica receptor cells of *A. longipes* (*n* = 27) fits well into this range. Like in other Tettigoniidae, the number of crista acustica cells decreases in the midleg and the hindleg (Houtermans and Schumacher 1974). The central projection of auditory fibres has a typical arrangement in the prothoracic ganglion. The fibers project into the auditory neuropile and terminate at the midline. It can be presumed that the crista acustica cells have a tonotopic projection like in other Tettigoniidae (Oldfield 1982). Thus, the neuroanatomy of this first-described Hetrodinae is in accordance with that of other Tettigoniidae (Lakes and Schikorski 1990).
